# 3D Face Modeling Using the Multi-Deformable Method

**DOI:** 10.3390/s121012870

**Published:** 2012-09-25

**Authors:** Jinkyu Hwang, Sunjin Yu, Joongrock Kim, Sangyoun Lee

**Affiliations:** Department of Electrical and Electronics Engineering, Yonsei University, 134 Shinchon-dong, Seodaemun-gu, Seoul 120-749, Korea; E-Mails: winispirit@yonsei.ac.kr (J.H.); biometrics@yonsei.ac.kr (S.Y.); jurock@yonsei.ac.kr (J.K.)

**Keywords:** 3D face modeling, face deformable model, face shape estimation, statistical face model

## Abstract

In this paper, we focus on the problem of the accuracy performance of 3D face modeling techniques using corresponding features in multiple views, which is quite sensitive to feature extraction errors. To solve the problem, we adopt a statistical model-based 3D face modeling approach in a mirror system consisting of two mirrors and a camera. The overall procedure of our 3D facial modeling method has two primary steps: 3D facial shape estimation using a multiple 3D face deformable model and texture mapping using seamless cloning that is a type of gradient-domain blending. To evaluate our method's performance, we generate 3D faces of 30 individuals and then carry out two tests: accuracy test and robustness test. Our method shows not only highly accurate 3D face shape results when compared with the ground truth, but also robustness to feature extraction errors. Moreover, 3D face rendering results intuitively show that our method is more robust to feature extraction errors than other 3D face modeling methods. An additional contribution of our method is that a wide range of face textures can be acquired by the mirror system. By using this texture map, we generate realistic 3D face for individuals at the end of the paper.

## 1. Introduction

Three-dimensional (3D) face modeling is a challenging topic in computer graphics and computer vision. Unlike 2D face models, 3D face models can realistically express face deformation and pose variation with depth information. With these advantages, 3D face models have been applied to various applications, including movies, 3D animation and telecommunications [1,2].

Three dimensional modeling systems can be categorized into active and passive vision systems [3]. An active vision system calculates 3D information by measuring a beam of light radiated from an external device such as a beam projector or laser. The typical 3D face modeling method using an active vision system constructs a 3D face mesh using a captured 3D point cloud [4–9]. In such systems, a 3D laser scanner or calibrated stereo camera with structured light can be used to capture 3D coordinates and texture information. While these methods are highly accurate, they are also time consuming, and the necessary equipment is expensive.

Nowadays, the passive vision-based 3D modeling system is preferred for human faces because the glare from light-emitting devices can be unpleasant for the users. Passive vision-based system means a system that needs no light-emitting devices and estimates 3D information from 2D images. In passive vision-based 3D face modeling, 3D information can be calculated by analyzing camera geometry from corresponding features in multiple views [10] or adjusting the statistical 3D face model to captured facial images [2,11]. For convenience, we call the former the corresponding feature-based 3D face modeling method and the latter the statistical model-based 3D face modeling method.

Among the 3D face modeling methods using the passive vision system, the most commonly used one is the corresponding feature-based 3D face modeling method. This method is less computationally expensive because it uses only a few feature points to generate a 3D facial shape. Additionally, this method can generate highly accurate 3D facial shapes by using real 3D information calculated from the camera geometry. However, the accuracy of the 3D facial shapes declines rapidly if the extracted locations of the corresponding points are not exact. This problem should be solved to apply to automatic 3D modeling system because even excellent feature extraction techniques such as the active appearance model (AAM) [12] and the active shape model (ASM) [13,14] can produce erroneous feature extraction results for indistinct parts of the face.

In this paper, we aim to develop a realistic 3D face modeling method that is robust to feature extraction errors and generates accurate 3D face modeling results. To achieve this, we propose a novel 3D face modeling method which has two primary steps: 3D facial shape estimation and texture mapping with a texture blending method.

In the 3D facial shape estimation procedure, we take a statistical model-based 3D face modeling approach as a fundamental concept. Among the statistical model-based methods, we use in particular the deformable face model that utilizes location information of facial features in the input image. This method is robust to feature extraction errors because it uses pre-trained 3D face data to estimate 3D facial shapes from input face images but it is a little less accurate than the corresponding feature-based 3D face modeling methods. To improve accuracy of the 3D facial shapes, we propose a 3D face shape estimation method using multiple 3D face deformable models.

In the texture mapping procedure, we apply a cylindrical mapping and a stitching technique to generate a texture map. When stitching each face part, we apply a modified gradient-domain blending technique [15] to remove the seam that appears at the boundaries of each face part because of photometric inconsistency.

This paper is organized as follows: in Section 2, we introduce previous 3D face modeling techniques and the 3D face deformable model that is basis of proposed method. In Section 3, we address our 3D facial shape estimation method with mirror system. In Section 4, we describe our texture mapping and texture map generation method using a modified gradient-domain blending technique. Then, we discuss the 3D face modeling results and evaluate our method's performances compared with those of other 3D face modeling methods and ground truth in Section 5. Finally, we conclude our paper and address future work in Section 6.

## 2. Preliminary Study

In this section, we address previous works and the fundamental concept of the proposed 3D face modeling method. In Sections 2.1 and 2.2, we introduce previous 3D face modeling methods using passive vision systems. We categorize them into two groups: corresponding feature-based 3D face modeling and statistical model-based 3D face modeling. Then, we study strengths and weaknesses of these methods. In Section 2.3, we concretely describe the 3D face modeling method using a deformable model which is a type of statistical model-based 3D face modeling because it is a fundamental concept of the proposed method that will be described in Section 3.1.

### 2.1. Corresponding Feature-Based 3D Face Modeling

The simplest and fastest way to generate a 3D face model using the corresponding features is to use orthogonal views [16–18]. In this method, the 3D coordinates of the features can be easily calculated from manually selected feature points in two orthogonal views of the face. This method is quite easy to implement, but orthogonality between the two views is necessary.

Some researchers construct 3D faces from several facial images. Fua *et al.* [19] proposed a regularized bundle-adjustment on triplet images to reconstruct 3D face models from image sequences. Their method takes advantage of a rough knowledge of the head's shape (from a generic face model), but it is computationally expensive because it requires dense stereo matching. In a similar approach, Lee *et al.* [20] constructed a 3D head model using two-pass bundle adjustments. In the first pass, the method computes several feature points of a target 3D head and then uses these features to obtain a roughly matched head model by modifying a generic head. Next, the second pass bundle adjustment is carried out to obtain a detailed 3D head model. Pighin *et al.* [21] developed a method that generates 3D faces by fitting a generic 3D face model on pre-defined 3D landmark points which are reconstructed from sequentially captured facial images. The pre-defined 3D landmark points can be calculated by the structured from motion (SfM) method after extracting the corresponding feature points in images captured from different views. The texture map is generated by combining the multi-view photographs into a cylindrical map. In their method, face images from different views provide a wide range of textures and features (e.g., the ears) which can be used to generate a realistic 3D face. However, the method is somewhat inconvenient because the user must hold a stationary pose during image capture. In addition, the 3D reconstruction is quite sensitive to the accuracy of the feature extraction so that a manually intensive procedure is required.

As another example of a corresponding feature-based method, Lin *et al.* [22] proposed a 3D face modeling system using two mirrors positioned next to the face to simultaneously capture three different views of the face. Then, they reconstruct 3D points annotated with markers by analyzing the relation between directly captured markers and the markers reflected in the mirrors. In their method, a wide range of face data can be acquired from the captured face images. In addition, this approach avoids the problem of synchronization of multiple cameras, although it is still sensitive to feature extraction error.

### 2.2. Statistical Model-Based 3D Face Modeling

In 3D face modeling using statistical model, the 3D morphable face model suggested by Blanz and Vetter [23] is the most well-known. To generate a 3D morphable face model, they construct a database including the 3D coordinates and skin texture from a real human face captured by a 3D laser scanner. Then, statistical analysis is carried out to determine control parameters for the 3D face shape and skin texture deformation. During the modeling procedure, the model parameters are iteratively adjusted in order to fit the model to the input image. This gives remarkably realistic results, but the computational cost is very high.

To improve the speed, researchers have proposed 3D face modeling methods using a single-view image. Kuo *et al.* [24,25] proposed a method to synthesize a lateral face from a single frontal-view image. They construct a facial image database containing both frontal and lateral views and then define anthropometric parameters that represent the distance between two features manually extracted by anthropometric definition. In the modeling stage, they estimate the lateral facial parameters from the input frontal image using the relationship between the frontal and lateral facial parameters. Baek *et al.* [26] suggested an anthropometry-based 3D face modeling technique. They built a database after measuring anthropometric information from anatomically meaningful 3D points among a 3D point cloud captured by a 3D laser scanner. Then, they created a statistical model to control the overall 3D face shape after statistical analysis of the database. This method is much faster than the 3D morphable face model, but the depth estimation results are sensitive to head poses, which may result in inaccurate distance measurement between landmarks. Importantly, this approach is also limited to the reconstruction of frontal views.

### 2.3. 3D Face Shape Model Generation

The 3D face deformable model is a type of parametric model that can deform shapes and textures by changing related parameters. The morphable face model [27], which generates a textured 3D face by controlling parameters that can be acquired from statistical analysis of 3D face scans containing geometric and textural data, is the most representative model in this class.

Generally, the computational costs of morphable face models are very high because they use entire face data (vertices and texture) and require many parameters to fit on input face images. On the other hand, a 3D face deformable model is less computationally expensive because it uses only geometric information (*i.e.*, the 3D coordinates of 3D face scans) and is composed of sparsely distributed vertices rather than full vertices of 3D scans. For clarity, the 3D deformable model that is composed of sparsely distributed vertices is called the 3D face shape model (FSM) in this paper. The 3D FSM is composed of anatomically meaningful 3D vertices which can represent the shape of the entire face. Each vertex is called a 3D FSM landmark, and a set of landmarks is called a 3D FSM face shape.

The deformation of 3D FSM can be carried out by global and local deformations. In a global deformation, the position and shape of the 3D FSM can be determined by a 3D affine transformation. The 3D affine transformation can be expressed as a 3 × 1 translation matrix (***T***) and a 3 × 3 matrix (***A***) of rotation and skew transformations. Under a global deformation, the coordinates of a vertex ([*X*,*Y*,*Z*]) can be transformed into ([*X_t_*,*Y_t_*,*Z_t_*]) by:

(1) $$\left[\begin{array}{c} X_{t}\\ Y_{t}\\ Z_{t} \end{array}\right]=\left[\begin{array}{ccc} A_{1} & A_{2} & A_{3}\\ A_{4} & A_{5} & A_{6}\\ A_{7} & A_{8} & A_{9} \end{array}\right]\;\left[\begin{array}{c} X\\ Y\\ Z \end{array}\right]+\left[\begin{array}{c} T_{1}\\ T_{2}\\ T_{3} \end{array}\right]$$

In a local deformation, a detailed deformation of 3D FSM can be parameterized by statistical analysis. First, 3D face shape data sets are constructed from 3D vertices corresponding to the position of 3D FSM landmarks in each 3D face scan. These sets (**S**) can be represented as:

(2) $$\begin{array}{l} {\text{S}}_{i}={\left[x_{1},y_{1},z_{1},x_{2},y_{2},z_{2},\cdots ,x_{N},y_{N},z_{N}\right]}^{T}\\ \text{S}={\left[{\text{S}}_{1},{\text{S}}_{2},\cdots ,{\text{S}}_{M}\right]}^{T} \end{array}$$

where *M* is the total number of face shape sets and *N* is the number of landmarks of 3D FSM. After constructing the 3D face shape data sets, a shape alignment procedure is carried out using 3D procrustes analysis [28], and the mean face shape is calculated as:

(3) $$\begin{array}{l} \bar{\text{S}}=\frac{1}{M}\sum_{i=1}^{M}{\text{S}}_{\text{i}} \end{array}$$

Then, feature vectors of the face data sets are calculated by principal component analysis (PCA). In PCA, the feature vectors of the shape data sets are the eigenvectors (**Φ**) of the covariance matrix (**Σ**) of the normalized shape data set (**D**):

(4) $$\begin{array}{l} \Gamma_{i}={\text{S}}_{\text{i}}-\bar{\text{S}}\\ \text{Σ}=\text{D}\cdot {\text{D}}^{\text{T}},\text{D}=\left[\Gamma_{1},\Gamma_{2},\cdots ,\Gamma_{\text{M}}\right]\\ \text{Σ}\cdot \text{Φ}=\text{Φ}\cdot \text{Λ} \end{array}$$

where Λ denotes a diagonal matrix of eigenvalues:

(5) $$\begin{array}{l} \text{Λ}=\left[\begin{array}{ccc} \lambda_{1} & & \\ & \ddots & \\ & & \lambda_{3M} \end{array}\right] \end{array}$$

Then, the local deformation can be parameterized with model parameter (*b*) and the sample mean of 3D face shape data (**S̄**). Finally, new 3D coordinates can be generated by the following equation:

(6) $$\hat{\text{S}}=\bar{\text{S}}+\sum_{i=1}^{m}{\text{Φ}}_{i}b_{i}$$

where *m* is the number of the modes of feature vectors.

**Figure 1.**
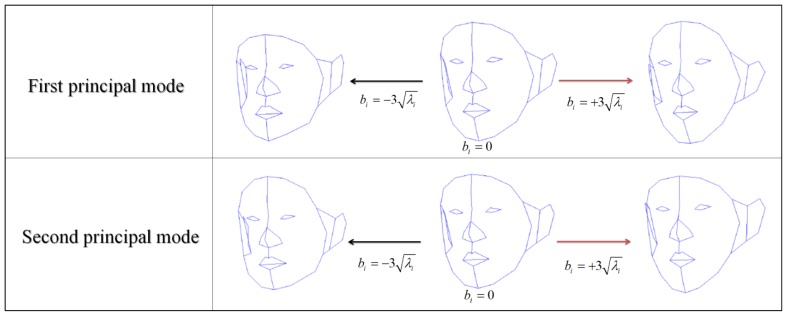
3D FSM deformation using model parameters of first and second principal mode. Deformation results applying a first principal mode parameter (**up**). Deformation results applying a second principal mode parameter (**bottom**).

In Equation (6), the local deformation of 3D FSM depends on only the model parameter (*b*) because the other variables are fixed. Therefore, determining the model parameter is a key to local deformations. Figure 1 shows deformation result of 3D FSM when the model parameters of first and second principal mode are changed.

## 3. 3D Facial Shape Estimation

In this section, we improve our proposed 3D face shape estimation method using a mirror system that was introduced at our previous work [29]. In the 3D face shape calculation using the 3D FSM, to use only the frontal face image may produce an inaccurate 3D face shape result that is different from the objective face shape because of uncertainty in the depth direction. To solve the problem, we calculate the 3D face shape by fitting multiple 3D FSMs to face images in three views. The multiple 3D FSMs include two virtual 3D FSMs which are applied to lateral face images and an original 3D FSM which is applied to a frontal face image. The virtual 3D FSMs can be generated by transforming the original 3D FSM symmetrically onto the mirror plane. After calculating the 3D face shape, detailed 3D face shapes can be interpolated by a generic 3D face model.

### 3.1. 3D Face Shape Estimation Using Multiple 3D FSMs in the Mirror System

**Figure 2.**
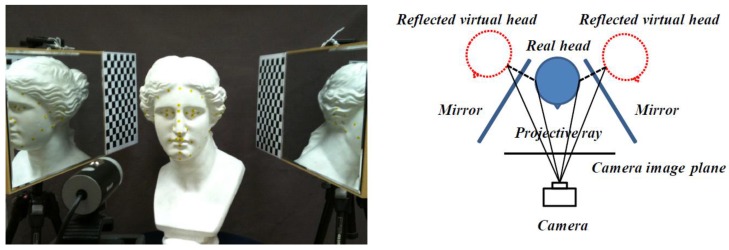
A mirror system for capturing facial images and a conceptual diagram of the system.

Our proposed face modeling system consists of two mirrors placed on either side of the face and a camera in front of the face. Frontal and lateral face images are captured simultaneously, and the pre-defined feature points are extracted from the captured image as described in Figure 2.

After feature extraction, the 3D FSM fitting procedure is carried out to calculate 3D coordinates from the extracted 2D feature points. During the fitting procedure, the 3D FSM parameters are adjusted to match the landmarks of the 3D FSM with the extracted feature points. This can be thought as least square optimization problem, and then the sum of the distances between the projected landmarks and objective feature points can be the cost function to be minimized. This cost function can be represented as:

(7) $$\begin{array}{l} F={\left\|{\text{S}}_{\text{Obj}}-{\text{S}}_{\text{ProjFSM}}\right\|}^{2}\\ {\text{S}}_{\text{Obj}}={\left[x_{1},y_{1},x_{2},y_{2},\cdots ,x_{N},y_{N}\right]}^{T}\\ {\text{S}}_{\text{ProjFSM}}={\left[{x^{\prime }}_{1},{y^{\prime }}_{1},{x^{\prime }}_{2},{y^{\prime }}_{2},\cdots ,{x^{\prime }}_{N},{y^{\prime }}_{N}\right]}^{T} \end{array}$$

where *N* is the number of landmarks of 3D FSM, **S***_Obj_* is the set of objective feature points in the image, and **S***_ProjFSM_* is the set of 3D FSM landmarks projected to the image plane.

The extracted feature points are categorized into three groups, **S***_FObj_*, **S***_LObj_* and **S***_RObj_* depending on the direction of the face. Then, three cost functions can be generated by the following equations:

(8) $$\begin{array}{l} F_{\text{Front}}={\left\|{\text{S}}_{\text{FObj}}-{\text{S}}_{\text{FProjFSM}}\right\|}^{2}\\ F_{\text{Left}}={\left\|{\text{S}}_{\text{LObj}}-{\text{S}}_{\text{LProjFSM}}\right\|}^{2}\\ F_{\text{Right}}={\left\|{\text{S}}_{\text{RObj}}-{\text{S}}_{\text{RProjFSM}}\right\|}^{2} \end{array}$$

The total cost function is then the sum of these three variables:

(9) $$\begin{array}{l} F_{\text{Total}}=F_{\mathrm{Front}}+F_{\mathrm{Left}}+F_{\mathrm{Right}} \end{array}$$

**Figure 3.**
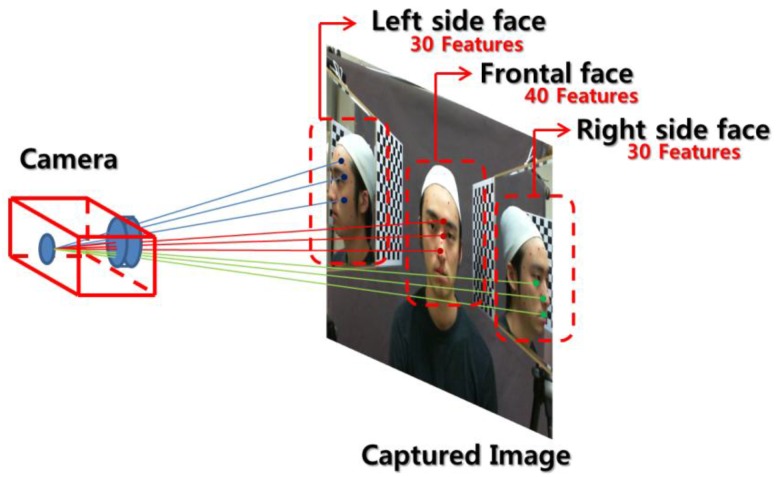
Feature extraction in simultaneously captured images and cost function generation.

In practice, 30 left side face features are extracted, while features like the right ear, right eye, *etc.*, remain occluded. Then, a cost function for left side face is determined as described in Equation (8). Next, 30 right side face features are extracted while the left side features like left ear, left eye, *etc.* are occluded, and a cost function can be determined like in the left side face case. In the frontal face case, only features of the ears are occluded, so we extract 40 features as described in Figure 3.

Meanwhile, **S***_FProjFSM_* can be directly calculated from the perspective projection of 3D FSM in Equation (8). However, additional calculations with respect to the mirror reflection are required to acquire **S***_LProjFSM_* and **S***_RProjFSM_*.

In mirror geometry, the mirror image of an object can be explained by the projection of a virtual 3D object reflected by the mirror plane onto the image plane, as described in Figure 2. A virtual 3D face can be generated by transforming the objective face symmetrically onto the mirror plane. Therefore, the location and orientation of the mirror planes should be calculated first.

An ideal, perfectly flat mirror plane can be represented by:

(10) aX+bY+cZ+d=0

where (*a*,*b*,*c*) is the normal vector of the mirror plane, and (*X*,*Y*,*Z*) are the 3D coordinates of an arbitrary point on the mirror plane. Then, the unknown coefficients *a*,*b*,*c* and *d* can be easily calculated by solving a linear equation using singular value decomposition (SVD). The 3D points on the mirror plane can be obtained by Zhang's camera calibration method [30] after attaching checkerboards to the mirrors.

**Figure 4.**
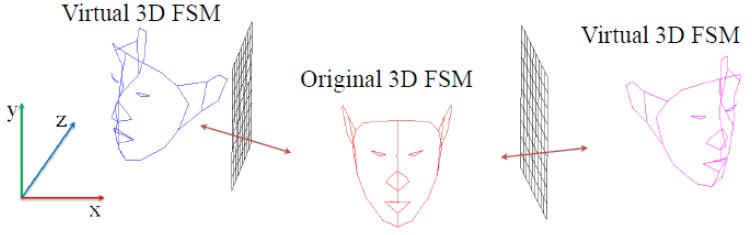
3D FSM and two virtual 3D FSMs generated by a Householder reflection.

Once the plane equation is calculated, a virtual 3D face can be generated using a Householder reflection. Given the 3D FSM landmarks (**P***_real_*) and the Householder matrix (**H***_u_*), the virtual 3D face can be calculated by:

(11) $$\begin{array}{l} {\text{P}}_{\text{img}}={\text{H}}_{u}{\text{P}}_{\mathrm{real}}-{\text{H}}_{u}{\text{P}}_{\mathrm{plane}}+{\text{P}}_{\mathrm{plane}}\\ {\text{H}}_{u}=\left({\text{I}}_{3\times 3}-2{\text{uu}}^{T}\right)\\ \text{u}={\left[A,B,C\right]}^{T} \end{array}$$

where **u** is the normal vector of the mirror plane, **I**_3×3_ is the 3 × 3 identity matrix, and **P***_plane_* is an arbitrary point on the mirror plane. Figure 4 describes the landmarks of 3D FSM and virtual 3D FSMs generated by the Householder reflection, where the red dots in the center represent original 3D FSM landmarks, and the green and blue dots represent the virtual 3D FSM landmarks derived from both mirrors. The red and green grids represent the mirror planes.

After generating the virtual 3D FSMs, we can calculate **S***_LProjFSM_* and **S***_RProjFSM_* by perspective projection under the camera coordinate system shown in Equation (12):

(12) $$\left[\begin{array}{c} x^{\prime }\\ y^{\prime }\\ 1 \end{array}\right]=\left[\begin{array}{ccc} \alpha_{x} & 0 & c_{x}\\ 0 & \alpha_{y} & c_{y}\\ 0 & 0 & 1 \end{array}\right]\;\left[\begin{array}{cccc} 1 & 0 & 0 & 0\\ 0 & 1 & 0 & 0\\ 0 & 0 & 1 & 0 \end{array}\right]\left[\begin{array}{c} x\\ y\\ z\\ 1 \end{array}\right]$$

In Equation (12), *α* (*α_x_* and *α_y_*) is the scale parameter, and *c* (*c_x_* and *c_y_*) is the principal point with respect to the x and y axes. These parameters can be easily determined from the camera calibration result when we calculate the 3D points on the mirror plane using Zhang's method [30]. The second term on the left-hand side is the normalized perspective projection matrix.

After the total cost function (*F_total_*) is determined, we calculate optimal solution to minimize it to fit the 3D FSM on the input face image. This can be expressed as:

(13) $$\begin{array}{l} {\text{x}}^{*}={\text{argmin}}_{x}\{F_{\mathrm{total}}\left(\text{x}\right)\}\\ \text{x}={\left[\hat{\text{A}},\text{T},\text{b}\right]}^{T} \end{array}$$

where **Â** is the column vector after stacking the elements of **A**. In global deformation, the unknown parameters are elements of the matrices **A** and **T**, and the vector **b** is an unknown parameter of local deformation.

To calculate the minimizer (x*), the iterative Levenberg-Marquardt optimization method is applied. In this method, in case of original 3D FSM, the partial derivatives of the Jacobian matrix can be easily calculated using the chain rule:

(14) $$\begin{array}{l} \begin{array}{l} F=\sum_{i=1}^{n}{\left(f_{i}\left(x\right)\right)}^{2},\text{f}=\left({\text{S}}_{\text{Obj}}-{\text{S}}_{\mathrm{ProjFSM}}\right)=\left[\begin{array}{ccc} f_{1} & \cdots & f_{n} \end{array}\right]\\ \text{J}\left(\text{x}\right)=\left[\begin{array}{ccc} \frac{\partial f_{1}}{\partial x_{1}} & \cdots & \frac{\partial f_{1}}{\partial x_{m}}\\ \vdots & \ddots & \vdots \\ \frac{\partial f_{n}}{\partial x_{1}} & \cdots & \frac{\partial f_{n}}{\partial x_{m}} \end{array}\right],\text{x}={\left[\hat{\text{A}},\text{T},\text{b}\right]}^{T}\\ \frac{\partial f_{j}}{\partial A_{i}}=\left({\frac{\partial f_{j}}{\partial \left(X_{t},Y_{t},Z_{t}\right)}|}_{P\left(X,Y,Z\right)}\right)\cdot \frac{\partial P\left(X,Y,Z\right)}{\partial A_{i}} \end{array}\\ \frac{\partial f_{j}}{\partial T_{i}}=\left({\frac{\partial f_{j}}{\partial \left(X_{t},Y_{t},Z_{t}\right)}|}_{P\left(X,Y,Z\right)}\right)\cdot \frac{\partial P\left(X,Y,Z\right)}{\partial T_{i}}\\ \frac{\partial f_{j}}{\partial b_{i}}=\left({\frac{\partial f_{j}}{\partial \left(X_{t},Y_{t},Z_{t}\right)}|}_{P_{t}\left(X,Y,Z\right)}\right)\cdot \left({\frac{\partial P_{t}\left(X,Y,Z\right)}{\partial \left(X,Y,Z\right)}|}_{\sum_{i=1}^{m}S_{i}b_{i}}\right)\cdot \frac{\partial \sum_{i=1}^{m}S_{i}b_{i}}{\partial b_{i}} \end{array}$$

where *m* is the number of 3D FSM parameters, *n* is the dimension of residual **f**.

However, for the virtual 3D FSM, the partial derivatives are changed due to the Householder transformation terms. After applying Equation (11) to Equation (1), the partial derivatives of the residual *f* can be calculated as:

(15) $$\begin{array}{l} \frac{\partial f_{j}}{\partial A_{i}}=\left({\frac{\partial f_{j}}{\partial \left(X_{\text{img}}^{k},Y_{\text{img}}^{k},Z_{\text{img}}^{k}\right)}|}_{P_{\text{img}}}\right)\cdot \left({\frac{\partial P_{\text{img}}\left(X_{t},Y_{t},Z_{t}\right)}{\partial \left(X_{t},Y_{t},Z_{t}\right)}|}_{P}\right)\cdot \frac{\partial P\left(X,Y,Z\right)}{\partial A_{i}}\\ \frac{\partial f_{j}}{\partial T_{i}}=\left({\frac{\partial f_{j}}{\partial \left(X_{\text{img}},Y_{\text{img}},Z_{\text{img}}\right)}|}_{P_{\text{img}}}\right)\cdot \left({\frac{\partial P_{\text{img}}\left(X_{t},Y_{t},Z_{t}\right)}{\partial \left(X_{t},Y_{t},Z_{t}\right)}|}_{P}\right)\cdot \frac{\partial P\left(X,Y,Z\right)}{\partial T_{i}}\\ \frac{\partial f_{j}}{\partial b_{i}}=\left({\frac{\partial f_{j}}{\partial \left(X_{\text{img}},Y_{\text{img}},Z_{\text{img}}\right)}|}_{P_{\text{img}}}\right)\cdot \left({\frac{\partial P_{\text{img}}\left(X_{t},Y_{t},Z_{t}\right)}{\partial \left(X_{t},Y_{t},Z_{t}\right)}|}_{P_{t}}\right)\cdot \left({\frac{\partial P_{t}\left(X,Y,Z\right)}{\partial \left(X,Y,Z\right)}|}_{P}\right)\cdot \frac{\partial \sum_{i=1}^{m}S_{i}b_{i}}{\partial b_{i}} \end{array}$$

where *A_i_* and *T_i_* are the parameters of the 3D affine transformation, and *b_i_* is a model parameter.

**Figure 5.**
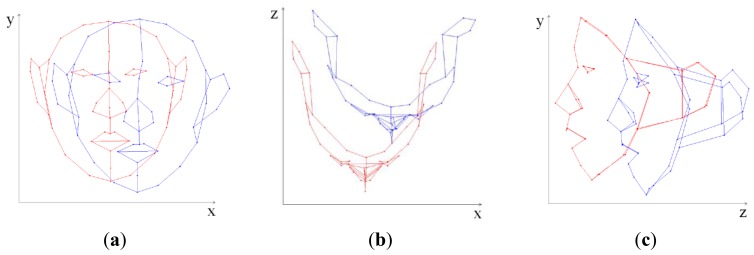
The 3D face shape estimation results. (**a**) Frontal view, (**b**) bird eye's view, (**c**) side view. The blue dots represent the landmarks of the initial 3D FSM, and the red dots represent the landmarks of the 3D face shape estimated by our method.

After calculating the Jacobian matrix about the three 3D FSM, the entire Jacobian matrix (**J***_All_*) can be reformulated by stacking each individual matrix. Then, the gradient of the cost function (∇*F*) can be calculated by:

(16) $$\begin{array}{l} {\text{J}}_{\text{All}}={\left[{\text{J}}_{F},{\text{J}}_{\text{LS}},{\text{J}}_{\text{RS}}\right]}^{T}\\ \nabla F={\text{J}}_{\text{All}}^{T}\text{f} \end{array}$$

where **J***_F_* is the Jacobian matrix from the front 3D FSM, **J***_LS_* is the Jacobian matrix of the virtual 3D FSM from the left mirror, and **J***_RS_* is the Jacobian matrix of the virtual 3D FSM from the right mirror. Figure 5 shows estimation results of the 3D FSM, where the blue dots represent the landmarks of the initial 3D FSM, and the red dots represent the landmarks of the 3D face shape estimated by our method.

### 3.2. Generic Model Fitting

After 3D face shape estimation, the 3D positions of other vertices can be determined by a generic 3D face model. The generic face model has been used in various applications because it has a uniform point distribution and can provide detailed face shape with a small number of points [3,4,26,31]. Among the previous approaches, the most common is a deformation technique based on the radial basis function (RBF) which can deform the vertices of the generic model by establishing the deformation function between the estimated 3D face feature points and the corresponding points of the generic model. Generally, the deformation function **P′** = *g*(**P**) takes the form of low order polynomial terms **M** and **t** added to a weighted sum of the radial basis function *ϕ*(*r*) with constraints Σ*_i_ w_i_* = 0 and Σ*_i_ w_i_***P***_i_* = 0:

(17) $$g(\text{P})=\sum_{i}w_{i}\phi \left(\left\|\text{P}-{\text{P}}_{i}\right\|\right)+\text{MP}+\text{t}$$

In Equation (17)**P***_i_* is a 3D feature point of the face, and P is a corresponding vertex of the generic model. To determine the weights of the radial basis function *w_i_* and the affine matrices **M** and **t**, Equation (18) is reformulated as linear equation **Ψ·X = Y**, where **Ψ**, **X** and **Y** can be expressed as follows:

(18) $$\begin{array}{l} \Psi =\left[\begin{array}{ccccccc} \phi \left(\left\|{\text{P}}_{1}-{\text{P}}_{1}\right\|\right) & \cdots & \phi \left(\left\|{\text{P}}_{1}-{\text{P}}_{\text{n}}\right\|\right) & p_{1x} & p_{1y} & p_{1z} & 1\\ \vdots & \ddots & \vdots & \vdots & \vdots & \vdots & \vdots \\ \phi \left(\left\|{\text{P}}_{\text{n}}-{\text{P}}_{1}\right\|\right) & \cdots & \phi \left(\left\|{\text{P}}_{\text{n}}-{\text{P}}_{\text{n}}\right\|\right) & p_{\mathrm{nx}} & p_{\mathrm{ny}} & p_{\mathrm{nz}} & 1\\ 1 & \cdots & 1 & 0 & 0 & 0 & 0\\ p_{1x} & \cdots & p_{2x} & 0 & 0 & 0 & 0\\ p_{1y} & \cdots & p_{2y} & 0 & 0 & 0 & 0\\ p_{1z} & \cdots & p_{2z} & 0 & 0 & 0 & 0 \end{array}\right]\in {\mathbb{R}}^{\left(n+4)\times (n+4\right)}\\ \text{X}={\left[\begin{array}{ccccc} w_{1} & \cdots & w_{n} & \text{M} & \text{t} \end{array}\right]}^{T}\in {\mathbb{R}}^{\left(n+4\right)\times 3}\text{Y}={\left[\begin{array}{ccccccc} {\text{q}}_{1} & \cdots & {\text{q}}_{\text{n}} & 0 & 0 & 0 & 0 \end{array}\right]}^{T}\in {\mathbb{R}}^{\left(n+4\right)\times 3} \end{array}$$

Once all of the parameters of the deformation function are determined, the vertices of the generic model can be deformed by multiplying **ψ̑** by **X**. Similar to **Ψ** in Equation (18), **ψ̑** can be established from other landmarks of the 3D FSM.

**Figure 6.**
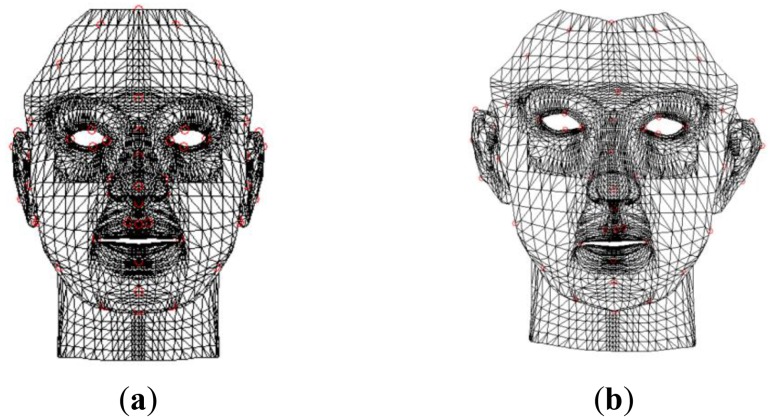
(**a**) Initial generic model and (**b**) deformed generic model after RBF interpolation.

Figure 6 shows the initial generic face model and deformed generic model after RBF interpolation. We edited the generic model created by Pighin [5] for use in our system. In Figure 6, the red circles in the generic model represent the 3D shape landmarks estimated in Section 2.2.

## 4. Texture Map Generation and Mapping

In this section, we introduce cylindrical mapping and address the stitching method using a seamless cloning method. A seam appears at the boundaries of each face part because of photometric inconsistency after stitching. To solve this problem, a seamless cloning method [15] with a gradient-domain blending technique is applied. This method successfully removed the seams at the boundaries, and thus a texture map of the whole face could be created.

### 4.1. Texture Extraction Using Cylindrical Texture Mapping

**Figure 7.**
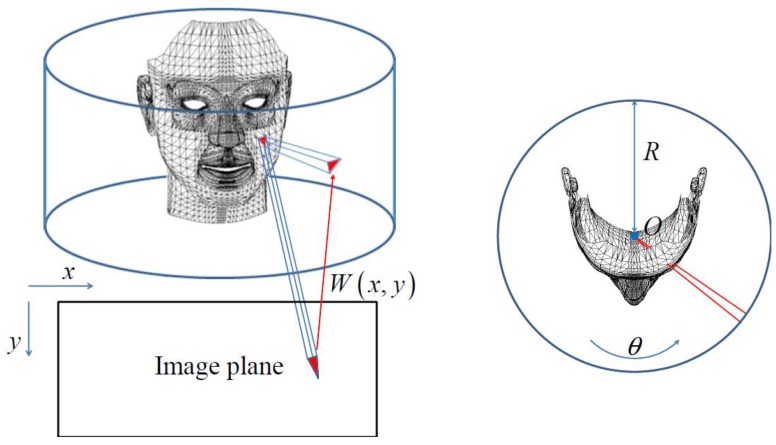
Cylindrical texture mapping for facial texture extraction.

To map textures on the 3D face model, a texture map is created by extracting the texture directly from the captured face image. For the sake of simplicity, cylindrical mapping is applied. In common cylindrical mapping methods, mesh vertices that are intersected with the ray passing through the center of a cylinder are projected onto the image plane after a virtual cylinder is placed around the 3D face model. Then, the colors of the corresponding pixels in the image are extracted and mapped to the texture map. However, this is time consuming because the positions of the vertices on the face mesh must be calculated. Thus, in our texture mapping procedure, vertices of a triangle mesh are projected onto the image plane, and then textures in the projected mesh are warped on the texture map, as shown Figure 7. Our system stitches together texture maps from the three different views to create a texture map of the entire face.

### 4.2. Texture Map Generation Using Modified Image Stitching Method

**Figure 8.**
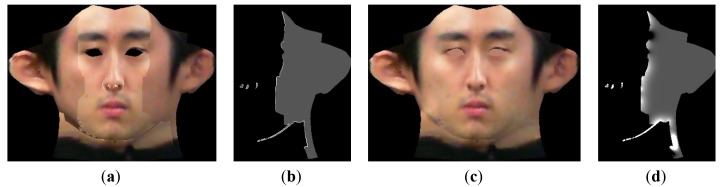
Texture map refinement using seamless cloning. (**a**) Face texture map after merging each face texture part, (**b**) initial map for seamless cloning, (**c**) synthesized face texture map after seamless cloning (**d**) *h*(*x*,*y*) map after 100 iterations of Equation (12).

As addressed in Section 4.1, a texture map of the entire face can be created by stitching each face texture parts. However, a seam appears at the boundaries of each face parts because of photometric inconsistency, as described in Figure 8(a). To solve this problem, a seamless image stitching method with gradient-domain blending techniques [15] is applied. Generally, gradient-domain blending techniques are more efficient at reducing photometric inconsistencies than is general image-domain blending. To implement the seamless cloning method, the entire texture image is first divided into three parts along the boundaries. Then, overlapping regions are created by expanding both parts by 1 pixel at the encountering region. Next, a *h*(*x*,*y*) map is created that is the same size as the original texture map of the whole face.

For initialization of the *h*(*x*,*y*) map, the side facial region having 0 pixel values is changed to have 1 pixel values. The pixel values in the overlapping regions are set to the ratio of the pixel value of the front view (*g*) to the pixel value of the side view (*f*):

(19) $$\begin{array}{l} h\left(x,y\right)=\{\begin{array}{cc} 1 & \begin{array}{c} g(x,y)=0\\ \mathrm{or}(x,y)\in R \end{array}\\ \frac{f(x,y)}{g(x,y)} & \text{otherwise} \end{array} \end{array}$$

where *R* is the side region.

The *h*(*x*,*y*) value is iteratively updated by calculating the solution of Laplace's equation at the corresponding pixel:

(20) $$h^{n+1}\left(x,y\right)=\frac{1}{4}\left(h^{n}\left(x+1,y\right)+h^{n}\left(x-1,y\right)+h^{n}\left(x,y+1\right)+h^{n}\left(x,y-1\right)\right)$$

Figure 8(b,d) shows an initial *h*(*x*,*y*) map and the resulting *h*(*x*,*y*) map after 100 iterations. In Figure 8(b), the *h*(*x*,*y*) values at the boundary are propagated into the valid area. After sufficient iterations, the final pixel values on the side of the face are calculated as the product of the updated *h*(*x*,*y*) and original pixel value, which allows for creation of a continuous texture map at the boundary:

(21) $$\hat{f}\left(x,y\right)=h^{n}(x,y)\cdot g\left(x,y\right)$$

**Figure 9.**
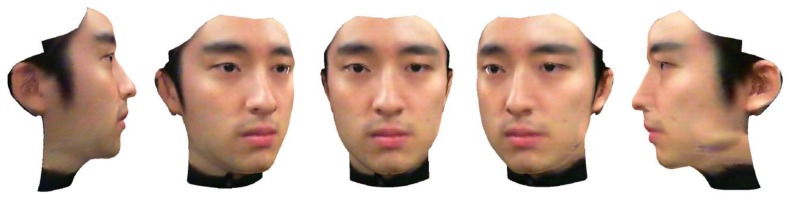
3D face modeling result after texture mapping.

After using morphological operations to fill in holes, a final face texture map can be created, as shown in Figure 8(c). Figure 9 shows the final 3D face after refining the texture map and the addition of artificial eyes.

**Figure 10.**
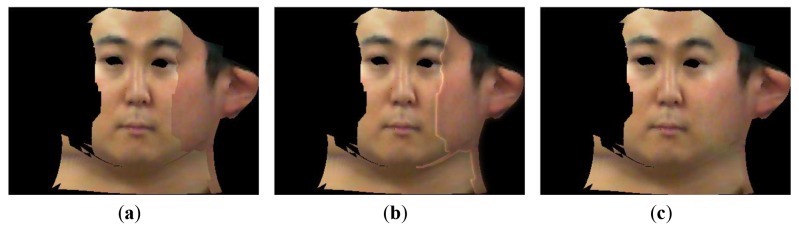
Image stitching results using multi-resolution splining and our method. (**a**) Image stitching without any blending methods. (**b**) Image stitching using multi-resolution splining. (**c**) Image stitching using our method.

Generally, multi-resolution splining [32] is well known as a blending method that operates well when the overlapped regions between images to be splined are broad enough. In our system, the overlapped region between each face part is only one pixel wide, therefore we couldn't get a satisfactory result when using multi-resolution splining method as described in Figure 10(b). On the other hand, the gradient domain image stitching method that is applied to our system shows excellent performance in spite of the narrow overlapped region as described in Figure 10(c). Figure 10 shows that the seam between boundaries of texture parts as described in Figure 10(a) is completely removed, while the seam remains after applying multi-resolution splining.

## 5. Experiments and Results

### 5.1. Experimental Settings

Before constructing the proposed face modeling system, we completed statistical analyses with respect to the 3D scan landmarks in order to calculate the feature vector elements of the local deformation parameters in the FSM, as described in Section 2.1. For the statistical analysis, we used principal component analysis (PCA). We recorded 3D face views of 100 individuals with a *Cyberware^TM^* laser scanner and then selected 50 landmarks in each face. During the PCA procedure, we retained 90% of the eigenvalue energy spectrum to reduce computational complexity.

**Figure 11.**
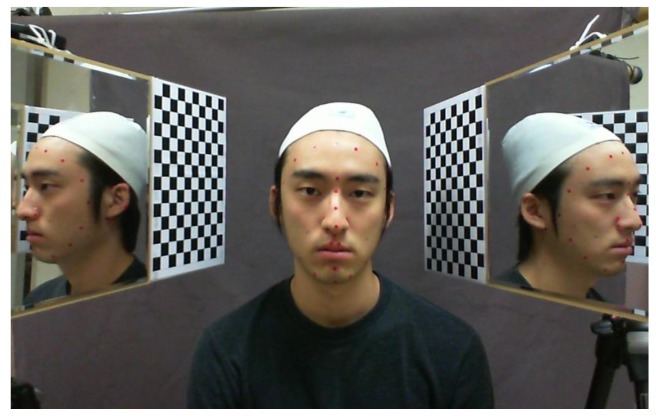
The user's face captured with our mirror system. The red color markers are attached on user's face for performance test.

To define the ground truth, we captured 3D faces of 30 individual with a 3D laser scanner at the same time that we captured the image with our proposed system. We attached color markers on each user's face to identify the feature points as shown in Figure 11 and then manually measured their 3D coordinates.

For a relative comparison, we used Lin's method [9]. That work is a good reference to evaluate the performance of our method because their mirror-based face modeling system is similar to ours. Additionally, we can indirectly compare our method and ordinary 3D reconstruction methods using epipolar geometry because they already compared their work with ordinary 3D reconstruction methods using epipolar geometry.

### 5.2. Accuracy Tests

We implemented Lin's method [22] and calculated the 3D coordinates of the marked feature points. In Lin's method, only the 3D coordinates of visible features can be reconstructed, so we compared the 3D reconstruction results of the 40 features on the frontal face with the actual faces. To compare the results with the actual faces, we aligned the 3D points from both methods with the 3D points of the actual faces. 3D procrustes analysis [28] was used to align the 3D coordinates of the reconstructed points. After aligning the 3D points, we calculated the average sum of the Euclidean distances between each point on the reconstruction and the actual faces, as described in Equation (22):

(22) $$\begin{array}{l} E=\frac{1}{M}\sum_{i=1}^{M}{\left\|S_{\text{Reconst}}-S_{\text{Ground}}\right\|}^{2}\\ {\text{S}}_{\text{Reconst}}={\left[x_{1},y_{1},z_{1},x_{2},y_{2},z_{2}\cdots ,x_{N},y_{N},z_{N}\right]}^{T}\\ {\text{S}}_{\text{Ground}}={\left[{x^{\prime }}_{1},{y^{\prime }}_{1},{z^{\prime }}_{1},{x^{\prime }}_{2},{y^{\prime }}_{2},{z^{\prime }}_{2}\cdots ,{x^{\prime }}_{N},{y^{\prime }}_{N},{z^{\prime }}_{N}\right]}^{T} \end{array}$$

Then, we compared the accuracy of their method with that of our method, as shown in Table 1, where our proposed method exhibits slightly higher absolute errors than that of Lin, but the standard deviation (Std) of our method was about two times lower.

### 5.3. Test on Robustness to Feature Extraction Error

To test on the robustness of our method with respect to feature extraction errors, we artificially generated erroneous feature points with normally distributed random distances and directions. Firstly, we calculated a two-dimensional matrix containing normally distributed random numbers using the Box-Muller method. Then, we generate the noisy feature points by adding each column vector of the matrix to the 2D coordinates of the feature points in the input face image. The feature points on the face for measurement were annotated by color markers. We assumed that the 2D positions of the marked feature points were the reference position.

We first tested the results of our proposed method and Lin's method according to error strength, which can be adjusted by changing the standard deviation of the random numbers. Table 2 shows the maximum error distances according to the standard deviation.

**Figure 12.**
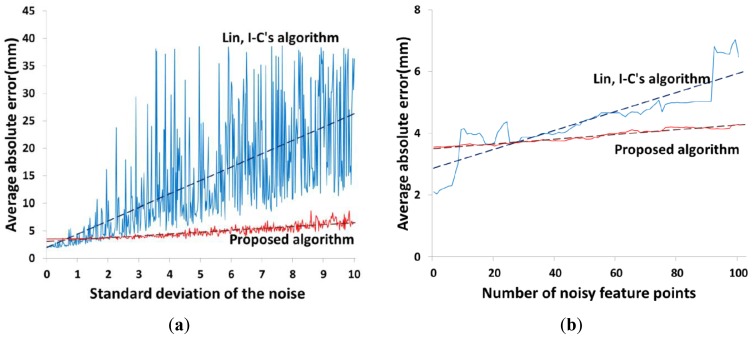
Robustness test results. (**a**) Error measurement results with increasing error strength by changing standard deviation of normally distributed random numbers. (**b**) Error measurement results with increasing number of noisy feature points.

We carried out the 3D face shape reconstruction by applying the proposed method and Lin's method with noisy feature points. The standard deviation of the error was varied from 0 to 5 in intervals of 0.02. Then, we calculated the average sum of the Euclidean distances (average absolute error) between each 3D reconstruction point and the truth. As shown in Figure 12(a), the average absolute error of the proposed method increases monotonically, while the average absolute error of Lin's method increases and fluctuates much more rapidly.

Next, we fixed the standard deviation and measured the average absolute error as the number of noisy feature points was increased from 0 to 100 in intervals of 1. As shown in Figure 12(b), the results are similar to those of the first robustness test. The average absolute error of the proposed method increases monotonically, but the average absolute error of Lin's method increases and fluctuates much more rapidly.

**Figure 13.**
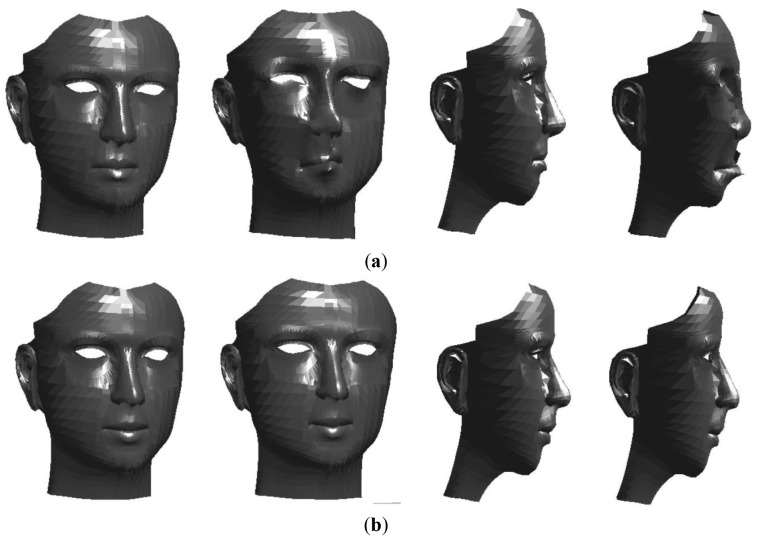
(**a**) 3D facial shape estimation results of Lin's method. (**b**) Proposed method with erroneous feature points.

Figure 13 shows the 3D face modeling results of proposed method and that of Lin with erroneous feature points. The standard deviation of the error is fixed at 3. As shown in Figure 13, the result of Lin's method shows a significantly distorted shape near the erroneous feature points. On the other hand, the proposed method maintains the overall face shape, even with the noisy feature points.

### 5.4. Textured 3D Face Model Generation Results

**Figure 14.**
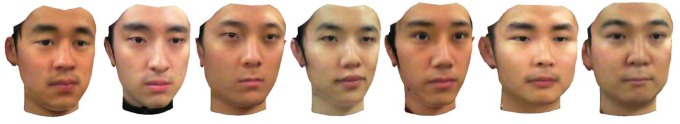
The results of the textured 3D face model for individuals.

We generated a textured 3D face model of users using the proposed face modeling method. After applying the generic model fitting as described in Subsection 3.2, we applied our texture mapping method described in Section 4. Eyeballs are not included in the generic model, and so we inserted artificial eyeballs with the 3D Max program. After producing the eyeballs, we align the center of the eyeball to the center of the eye region. Figure 14 shows the input face image, the texture map and the textured 3D face.

## 6. Conclusions

In this paper, we propose a realistic 3D face modeling method that is robust to feature extraction errors and can generate accurate 3D face models. In the facial shape estimation procedure, we propose a 3D face shape estimation method using multiple 3D face deformable models in a mirror system. The proposed method shows high robustness to feature extraction errors and highly accurate 3D face modeling results, as described in Sections 5.2 and 5.3. In the texture mapping procedure, we apply cylindrical mapping and stitching technique to generate a texture map. We apply the seamless cloning method, which is a type of gradient-domain blending technique, to remove the seam that caused by photometric inconsistency and finally can thus acquire a natural texture map.

To evaluate our method's performance, we carry out accuracy and robustness tests with respect to 30 individuals' 3D facial shape estimation results. Our method shows not only highly accurate 3D face shape results when compared with the ground truth, but also robustness to feature extraction errors. Moreover, the 3D face rendering results intuitively show that our method is more robust to feature extraction errors than other 3D face shape estimation methods. An additional contribution of our method is that wide range of face textures can be acquired by the mirror system. Lastly, we generate textured 3D faces using our proposed method. The results show that our method can generate very realistic 3D faces, as shown in Figure 14. Our ultimate goal is to create an automatic 3D face modeling system, and so we plan to apply automatic feature extraction processes to our method. This may be a problem for side view images and will require the development of new techniques that will be described in future works.

## Figures and Tables

**Table 1.** Mean, standard deviation and median of absolute distance errors of the proposed and Lin. I-C's method compared to the actual faces.

**Estimation Method**	**Absolute Error (mm)**		

	**Mean**	**Std**	**Median**
**Lin.'s method** [22]	3.12	1.14	2.59
**Proposed method**	3.58	0.59	3.49

**Table 2.** The maximum error distances according to the standard deviation.

**Standard deviation of the error**	1	2	3	4	5
**Maximum error distances (pixel)**	3.811	6.692	9.760	12.667	16.845
